# Supplementary material for: Mycotoxin Deoxynivalenol Has Different Impacts on Intestinal Barrier and Stem Cells by Its Route of Exposure
Source: Toxins (Basel). 2020 Sep 24;12(10):610. doi: 10.3390/toxins12100610 (PMC7598581; doi:10.3390/toxins12100610)
Supplement: Supplementary file 1 [file toxins-12-00610-s001.zip › toxins-936336 sp/Supplementary Materials/toxins-936336-supplementary-v2.pdf]

# Supplementary Materials: Mycotoxin Deoxynivalenol Has Different Impacts on Intestinal Barrier and Stem Cells by Its Route of Exposure

**Hikaru Hanyu, Yuki Yokoi, Kiminori Nakamura, Tokiyoshi Ayabe, Keisuke Tanaka, Kinuko Uno, Katsuhiro Miyajima, Yuki Saito, Ken Iwatsuki, Makoto Shimizu, Miki Tadaishi and Kazuo Kobayashi-Hattori**

## Supplemental Figures Tables and Videos

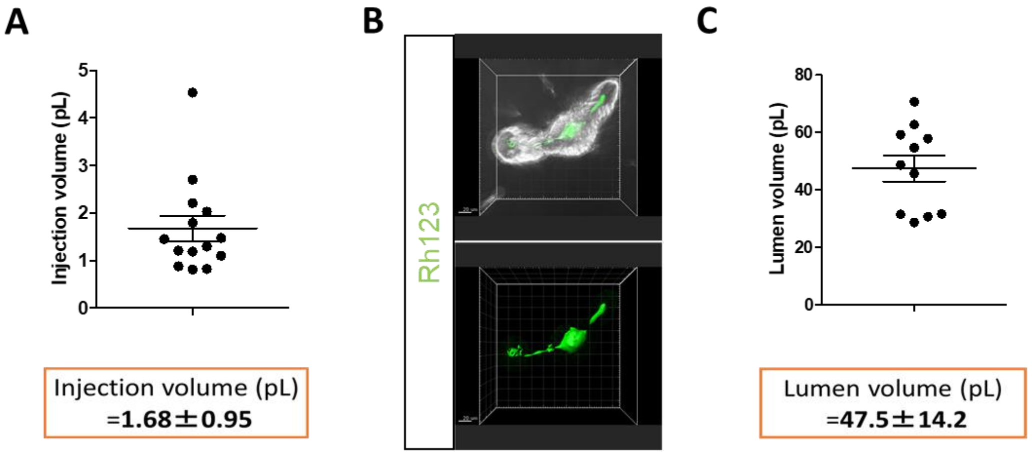


**Figure S1.** Measurement of the volume of injection solution and enteroid lumen. (**A**) Injection volume (pL) was calculated as previously described by Yokoi et al. [1]. Mean ± SEM; $n = 14$. (**B**) Representative 3-D image of enteroids cultured with 1 μM rhodamine 123 (Rh123; green) for 3 h. Scale bars: 20 μm. (**C**) Luminal volume was calculated from the 3-D image of enteroids using the Imaris 8 software. Mean ± SEM; $n = 11$.

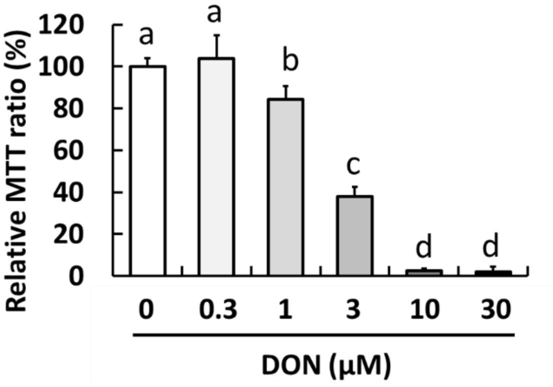


**Figure S2.** 3-(4,5-dimethylthiazol-2-yl)-2,5-diphenyltetrazolium bromide (MTT) assay in enteroids exposed to different doses of basolateral DON. Cell viability was investigated using enteroids treated with 0, 0.3, 1, 3, 10, or 30 μM DON through MTT assay. The cell viability of untreated organoids (0 μM DON) was defined as having 100% viability and that of the DON-treated enteroids was relatively expressed. Different lowercase letters indicate significant differences. Mean ± SE; $n = 5$ ($p < 0.05$, Tukey's post hoc test).

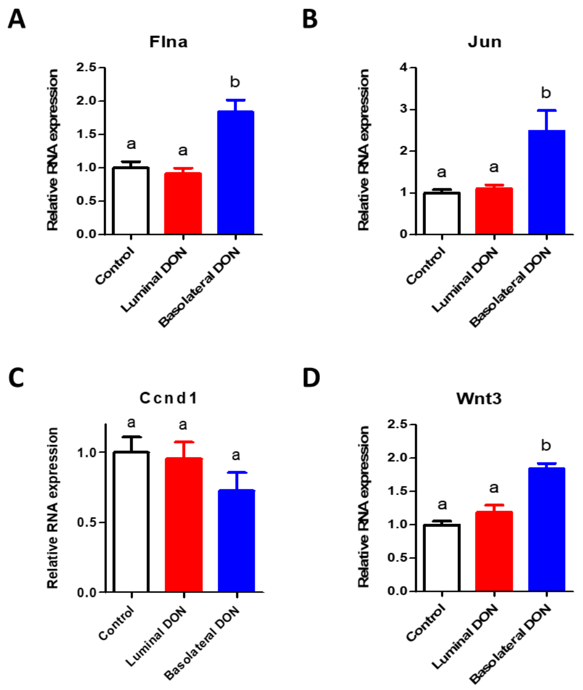


**Figure S3.** The mRNA levels of the key genes in Kyoto Encyclopedia of Genes and Genomes (KEGG) pathway analysis. Relative RNA expression of Flna (Filamin), Jun (c-jun), Ccnd1 (CycD), and Wnt3 (Wnt) in enteroids after 24 h treatment (control, 1 μM luminal DON exposure or 1 μM basolateral DON exposure) was measured through qPCR. Flna **(A)**, Jun **(B)**, and Ccnd1 **(C)** are the representative genes in the focal adhesion pathway (mmu04510). Flna **(A)** is one of the representative genes in mitogen-activated protein kinases (MAPK) signaling pathway (mmu04010). Wnt3 **(D)** is one of the representative gene in MAPK signaling pathway (mmu04310). Different lowercase letters indicate significant differences. Mean ± SEM; $n$ = 4 for the luminal DON exposure group and $n$ = 7 for the control and the basolateral DON exposure groups ($p < 0.05$, Tukey's post hoc test).

**A**

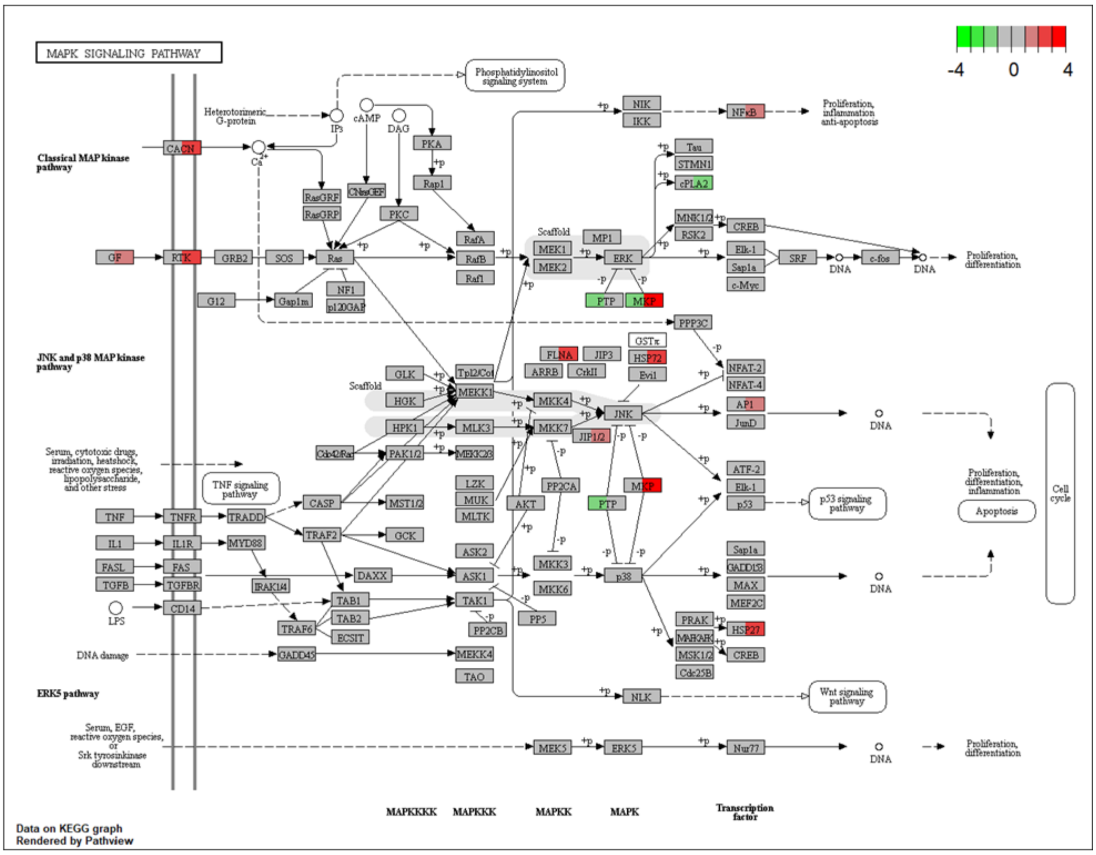


**B**

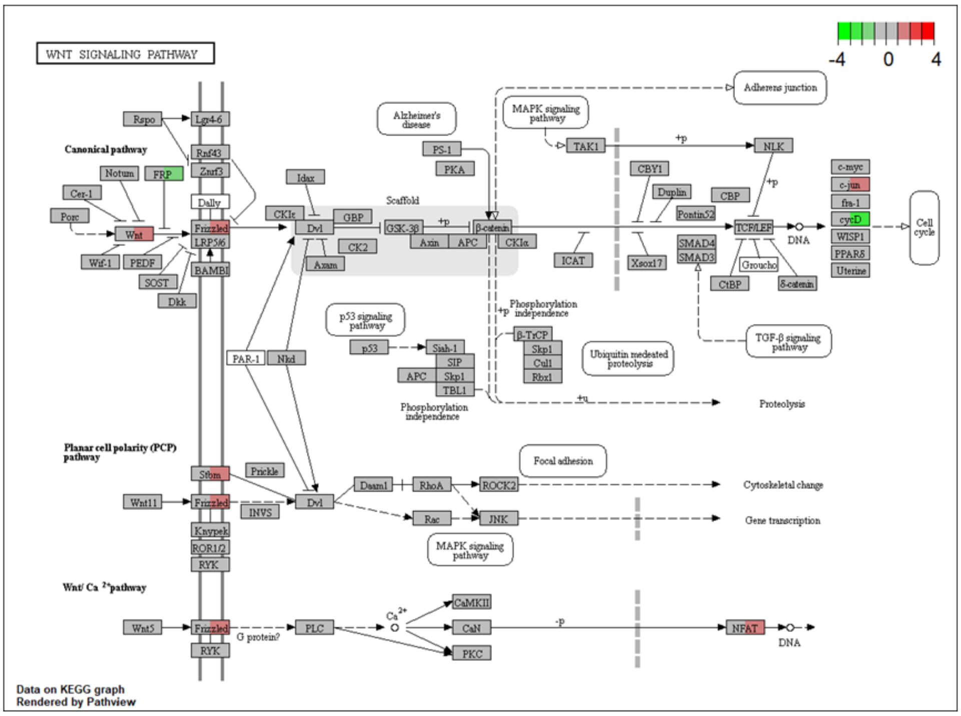


**Figure S4.** KEGG pathway analysis of enteroids after basolateral or luminal DON exposure. KEGG pathway of (**A**) the MAPK signaling pathway (mmu04010) or (**B**) Wnt signaling pathway (mmu04310). Green- or red-colored gene name boxes indicate downregulated or upregulated genes in enteroids exposed to luminal DON (left) or basolateral DON (right), compared with the control.

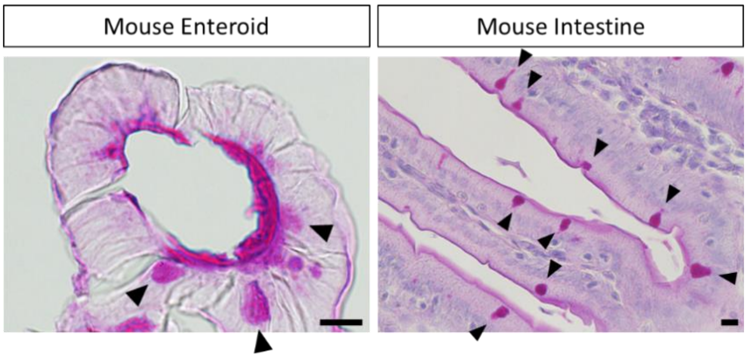


**Figure S5.** Periodic Acid–Schiff (PAS)-stained enteroids and small intestine tissues. Representative images of PAS-stained mouse enteroids (left) and mouse small intestine tissues (right). The nuclei in mouse small intestine tissues were stained with hematoxylin. Black arrowheads show goblet cells. Scale bars: 10 μm.

**Table S1.** Calculation of the dilution ratio.

| Volume of Enteroid Lumen (pL) | Injection Volume (pL) | Dilution Ratio |
|---|---|---|
| 47.5±14.2 | 1.68±0.95 | 28.3 |

**Table S2.** The database for annotation, visualization and integrated discovery (DAVID) enrichment analysis.

| Term | Count | % | P-Value | Fold Enrichment |
|---|---|---|---|---|
| mmu04510:Focal adhesion | 14 | 2.60223 | 0.001796 | 2.723373 |
| mmu04010:MAPK signaling pathway | 15 | 2.788104 | 0.003639 | 2.406395 |
| mmu00640:Propanoate metabolism | 5 | 0.929368 | 0.004057 | 7.456855 |
| mmu04151:PI3K-Akt signaling pathway | 18 | 3.345725 | 0.005946 | 2.064975 |
| mmu01100:Metabolic pathways | 46 | 8.550186 | 0.006178 | 1.45964 |
| mmu00830:Retinol metabolism | 8 | 1.486989 | 0.006286 | 3.619507 |
| mmu04390:Hippo signaling pathway | 10 | 1.858736 | 0.012325 | 2.66669 |
| mmu05145:Toxoplasmosis | 8 | 1.486989 | 0.014959 | 3.067963 |
| mmu00620:Pyruvate metabolism | 5 | 0.929368 | 0.015118 | 5.162438 |
| mmu00260:Glycine, serine and threonine metabolism | 5 | 0.929368 | 0.016482 | 5.033377 |
| mmu01130:Biosynthesis of antibiotics | 12 | 2.230483 | 0.016824 | 2.257963 |
| mmu01200:Carbon metabolism | 8 | 1.486989 | 0.024488 | 2.777036 |
| mmu05146:Amoebiasis | 8 | 1.486989 | 0.025522 | 2.7533 |
| mmu00072:Synthesis and degradation of ketone bodies | 3 | 0.557621 | 0.028837 | 10.98191 |
| mmu04915:Estrogen signaling pathway | 7 | 1.301115 | 0.033989 | 2.876215 |
| mmu04066:HIF-1 signaling pathway | 7 | 1.301115 | 0.040152 | 2.763423 |
| mmu04146:Peroxisome | 6 | 1.115242 | 0.054015 | 2.910869 |
| mmu04931:Insulin resistance | 7 | 1.301115 | 0.054495 | 2.562446 |
| mmu05222:Small cell lung cancer | 6 | 1.115242 | 0.056335 | 2.876215 |
| mmu04310:Wnt signaling pathway | 8 | 1.486989 | 0.059858 | 2.284653 |
| mmu04512:ECM-receptor interaction | 6 | 1.115242 | 0.06618 | 2.745478 |
| mmu04912:GnRH signaling pathway | 6 | 1.115242 | 0.06618 | 2.745478 |
| mmu04978:Mineral absorption | 4 | 0.743494 | 0.070916 | 4.12995 |
| mmu04020:Calcium signaling pathway | 9 | 1.672862 | 0.077591 | 2.013351 |
| mmu00220:Arginine biosynthesis | 3 | 0.557621 | 0.078874 | 6.35795 |
| mmu05031:Amphetamine addiction | 5 | 0.929368 | 0.0833 | 3.005001 |
| mmu04115:p53 signaling pathway | 5 | 0.929368 | 0.0833 | 3.005001 |
| mmu04380:Osteoclast differentiation | 7 | 1.301115 | 0.091444 | 2.237056 |

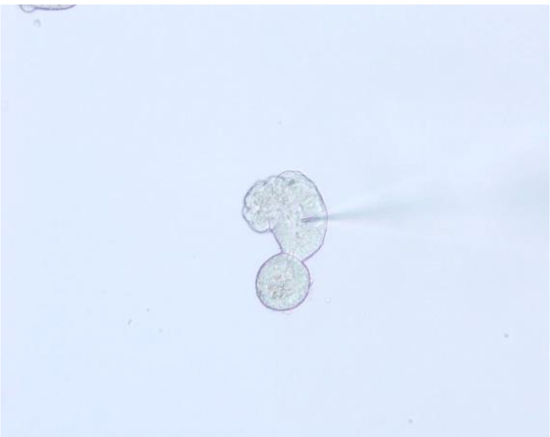

**Video S1.** Microinjection into enteroids. A representative movie of microinjection of solution into enteroids.

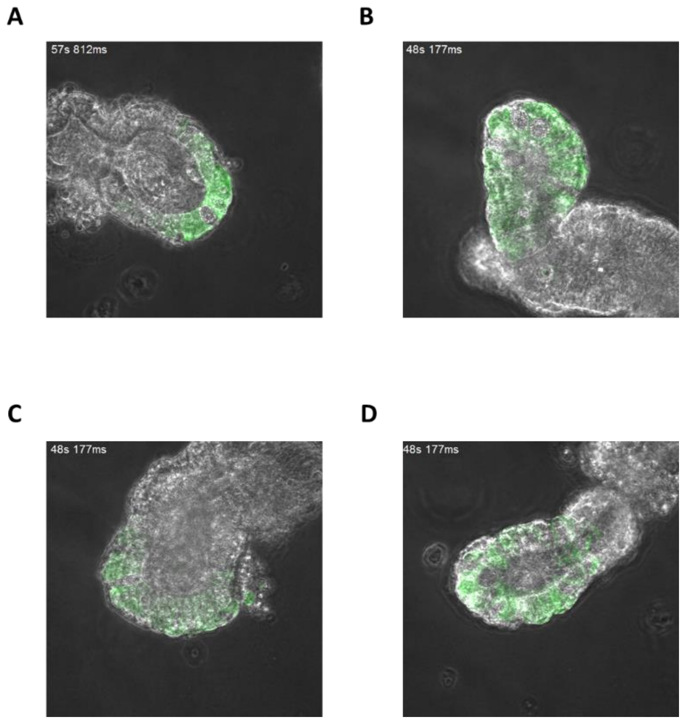


**Video S2.** Live imaging of Lgr5-EGFP enteroids after DON exposure**.** Representative movie segments of Lgr5-EGFP enteroids after DON exposures. (**A**) Control, (**B**) PBS-injected enteroids, (**C**) enteroids exposed to luminal DON, and (**D**) enteroids exposed to basolateral DON. Lgr5-EGFP$^{+}$ cells (green) show Lgr5$^{+}$ stem cells. These are supplemental movie segments of Figure 4A.

## Supplementary Materials and Methods

## 1. Measurement of Dilution Ratio During Microinjection into Enteroids

### *1.1. Injection Solution Volume*

Glass capillary (Femtochips II; Eppendorf AG, Hamburg, Germany) was adjusted to 3–4 µm inner diameter by breaking the tip with a microloader and filled with mineral oil and fluorescein isothiocyanate (FITC; Sigma-Aldrich Co. LLC, St. Louis, MO, USA). The inner diameter ($r_1$ and $r_2$) and moving distance (h) of the liquid interface between the mineral oil and FITC in the needle were measured under the microscope before and after microinjection. The injection volume was calculated using a previously described formula [1], as follows (1):

$$\text{The injection volume} = 1/12\pi(r_1^2 + r_1 r_2 + r_2^2) \times h \quad (1)$$

### *1.2. Enteroid Lumen Volume*

The enteroid lumen was stained with rhodamine123 (Rh123; Sigma–Aldrich Co. LLC, St. Louis, MO, USA), as previously described [1]. Briefly, enteroids on day 3 of culture were treated with 1 µM Rh123 for 3 h at 37 °C, and 1-µm Z-stack images were acquired using a confocal microscope (Fv10i; OLYMPUS Co.). The enteroids' luminal volume was measured as Rh123 volume incorporated into the enteroid lumen using a 3-D measurement tool, Imaris 8 software (Bitplane, Belfast, UK).

### *1.3. Dilution Ratio*

The dilution ratio of substances introduced into the enteroid lumen through microinjection was calculated as follows (Equation (2)):

$$\text{Dilution ratio} = (\text{enteroid lumen volume})/(\text{injection volume}) \quad (2)$$

## 2. MTT Cell Viability Assay

On day 3 of culture, the enteroids were transferred into 96-well cell culture plate (NIPPON Genetics Co., Ltd., Tokyo, Japan), and enteroid viability was assessed using 3-(4,5-di-methylthiazol-2-yl)-2,5-diphenyltetrazolium bromide (MTT) assay. The seeded enteroids' viability was normalized before DON exposure through incubation with resazurin, as previously described [2]. Briefly, the organoids were cultured with 10 μg/mL resazurin (Sigma-Aldrich Co. LLC, St. Louis, MO, USA) for 6 h and the supernatant was transferred into a black 96-well plate before measuring using a fluorescence detector (excitation 530 nm; emission 590 nm) (GE Healthcare, Chicago, IL, USA). Then, 500 μg/mL of MTT solution (Sigma–Aldrich Co. LLC, St. Louis, MO, USA) in distilled water (DW) was added to the enteroid culture medium after treatment with 0.3, 1, 3, 10, or 30 μM DON for 18 h in the enteroid culture medium (for basolateral exposure). The medium was discarded, and 20 μL of 2% SDS (Sigma–Aldrich Co. LLC, St. Louis, MO, USA) solution in DW was added to solubilize the Matrigel (2 h, 37 °C) after incubation for 2 h at 37 °C under 5% $CO_2$. Then, 150 μL of DMSO was added for 1 h (37 °C) to solubilize MTT, and the absorbance at 562 nm was measured with a microplate reader (GE Healthcare, Chicago, IL, USA). The Matrigel-containing wells without enteroids were used as control and defined as having 0% viability. The untreated organoids (0 μM DON) were defined as having 100% viability and that of the DON-treated organoids was relatively expressed.

## 3. The qPCR Analysis

The total RNA was isolated from the enteroids using ISOGEN II (Nippon Gene Co., Ltd., Tokyo, Japan), and the cDNA was synthesized using a PrimeScript™ RT reagent Kit with gDNA Eraser (Perfect Real Time; Takara Bio Inc., Shiga, Japan), according to the manufacturer's instructions. Real-time PCR was conducted in an ABI PRISM 7300 Sequence Detection System (Thermo Fisher Scientific Inc., Waltham, MA, USA) using THUNDERBIRD® SYBR® Green (TOYOBO Co., Ltd., Osaka, Japan). The PCR conditions were as follows: 30 s at 95 °C, 40 cycles of 5 s at 95 °C, and 30 s at 60 °C. The mRNA level of each gene was normalized with that of β2-microglobulin (B2m), the mRNA level of the control group was set to 1, and that of the target genes was relatively expressed. The primer sequences were as follows: Filamin, alpha (Flna) forward, 5′-ACTGTAAAGGGTCCCAGGTGAG-3′ and reverse, 5′-GACCAATGTTCTGGCCACCC-3′; Jun proto-oncogene (Jun) forward, 5′-GCACATCACCACTACACCGA-3′ and reverse, 5′-GGGAAGCGTGTTCTGGCTAT-3′; cyclin D1 (Ccnd1) forward, 5′-CATCAAGTGTGACCCGGACTG-3′ and reverse, 5′-CCTTGGGGTCGACGTTCTG-3′; Wnt3 forward, 5′- TGGAACTGTACCACCATAGATGAC-3′ and reverse, 5′- ACACCAGCCGAGGCGATG-3′; B2m forward, 5′-CTGGTGCTTGTCTCACTGACCG-3′ and reverse, 5′-GCAGTTCAGTATGTTCGGCTTCC-3′.

## 4. Periodic Acid–Schiff (PAS) Staining

### *4.1. PAS Staining of Enteroids*

Cryosectioning of enteroids was performed as previously described [3]. Briefly, enteroids in the Matrigel suspension were arranged like a dome in the six-well cell culture plate (NIPPON Genetics Co., Ltd., Tokyo, Japan). Each dome contains 50 μL of enteroids resuspended in Matrigel. The enteroid-containing domes were fixed with 4% cold paraformaldehyde (Nacalai Tesque, Inc., Kyoto, Japan) for 30 min at room temperature after matrix polymerization at 37 °C under 5% $CO_2$. The domes were collected using a spatula and immersed in 20% sucrose solution for three days after fixation. Once the domes reached the bottom of the wells, the domes were collected from the sucrose solution using forceps, put in a mold containing the optimal cutting temperature (OCT) compound (Sakura Finetek Japan Co., Ltd., Tokyo, Japan), and the mold was placed in liquid nitrogen before cryosectioning using a cryostat (Leica Biosystems, Wetzlar, Germany). The cryosections were stained as follows: (1) Washed OCT with DW, (2) incubated with 0.5% periodic acid solution for 5 min, (3) incubated with Schiff's reagent solution for 5 min, and (4) incubated with bisulfite solution for 3 min.

### *4.2. PAS Staining of Small Intestine Tissue*

The tissue was fixed with 3.7% formalin (Nacalai Tesque, Inc., Kyoto, Japan) for 24 h. The tissue was dehydrated and penetrated with paraffin using Tissue-Tek VIP 5 Jr (Sakura Finetek Japan Co., Ltd., Tokyo, Japan) after fixation, followed by immersion in 70% ethanol overnight. Then, the tissue was embedded in paraffin using Tissue-Tek TEC (Sakura Finetek Japan Co., Ltd., Tokyo, Japan), and the sections were prepared with a microtome (Yamato Kohki Industrial Co., Ltd., Saitama, Japan). The paraffin-embedded sections were stained as follows: (1) Deparaffinized with xylene, (2) hydrophilized with ethanol, (3) incubated with 0.5% periodic acid solution for 5 min, (4) incubated with Schiff's reagent solution for 15 min, and (5) incubated with bisulfite solution for 3 min (thrice) and then Mayer's hematoxylin solution for 1 min.

## References

1. Yokoi, Y.; Nakamura, K.; Yoneda, T.; Kikuchi, M.; Sugimoto, R.; Shimizu, Y.; Ayabe, T. Paneth cell granule dynamics on secretory responses to bacterial stimuli in enteroids. *Sci. Rep.* **2019**, *9*, 2710, doi:10.1038/s41598-019-39610-7.
2. Grabinger, T.; Luks, L.; Kostadinova, F.; Zimberlin, C.; Medema, J.P.; Leist, M.; Brunner, T. Ex vivo culture of intestinal crypt organoids as a model system for assessing cell death induction in intestinal epithelial cells and enteropathy. *Cell Death Dis.* **2014**, *5*, e1228–e1228.
3. Degese, S.; Benton, G. Immunofluorescence of organoids embedded in basement membrane matrix. Trevigen Technical Tips. 2017. https://resources.rndsystems.com/images/site/dw_organoidIfprotocol_34748-web.pdf (accessed on 1 September 2020)
